# Xenon Flash Lamp Lift-Off Technology without Laser for Flexible Electronics

**DOI:** 10.3390/mi11110953

**Published:** 2020-10-22

**Authors:** Sang Il Lee, Seong Hyun Jang, Young Joon Han, Jun yeub Lee, Jun Choi, Kwan Hyun Cho

**Affiliations:** 1 Manufacturing Process Platform R&D Department, Korea Institute of Industrial Technology (KITECH), Ansan 15588, Korea; twosangone@kitech.re.kr (S.I.L.); youngjhan@kitech.re.kr (Y.J.H.); june522@kitech.re.kr (J.y.L.); 2 Human Convergence Technology R&D Department, Korea Institute of Industrial Technology (KITECH), Ansan 15588, Korea; seonghyun@kitech.re.kr

**Keywords:** flexible display, xenon flash lamp (XFL), light to heat conversion layer (LTHC), lift-off

## Abstract

This study experimentally investigated process mechanisms and characteristics of newly developed xenon flash lamp lift-off (XF-LO) technology, a novel thin film lift-off method using a light to heat conversion layer (LTHC) and a xenon flash lamp (XFL). XF-LO technology was used to lift-off polyimide (PI) films of 8.68–19.6 μm thickness. When XFL energy irradiated to the LTHC was 2.61 J/cm^2^, the PI film was completely released from the carrier substrate. However, as the energy intensity of the XFL increased, it became increasingly difficult to completely release the PI film from the carrier substrate. Using thermal gravimetric analysis (TGA), Fourier-transform infrared spectroscopy (FTIR) and transmittance analysis, the process mechanism of XF-LO technology was investigated. Thermal durability of the PI film was found to deteriorate with increasing XFL energy intensity, resulting in structural deformation and increased roughness of the PI film surface. The optimum energy intensity of 2.61 J/cm^2^ or less was found to be effective for performing XF-LO technology. This study provides an attractive method for manufacturing flexible electronic boards outside the framework of existing laser lift-off (LLO) technology.

## 1. Introduction

As interest in wearable electronic devices grows, research into flexible displays, the core technology for next-generation displays, is being actively conducted [1,2,3]. Various plastic materials are mentioned as flexible display substrate materials, but polyimide (PI), which has excellent heat resistance, is mainly being used as the substrate material [4,5]. However, flexible substrates such as PI are difficult to fabricate with various high-trust and high-performance electronic device manufacturing processes such as high-resolution photography [6,7,8]. Accordingly, the flexible substrate is coated on a carrier substrate such as glass, and then the device is manufactured using existing manufacturing equipment and processes for the glass substrate. The flexible display manufacturing process is completed by separating the carrier substrate from the flexible substrate after all other element manufacturing processes have been performed.

The lift-off is one of the most important process steps and can have a decisive influence on the overall yield of flexible display elements [9,10]. Currently, lift-off is primarily performed using laser lift-off (LLO) technology with an excimer laser as the light source. LLO technology involves irradiating the excimer laser beam through an optical system and the glass carrier substrate surface and separating the PI film from the bonding surface of the carrier substrate [10]. This is similar to the excimer laser annealing (ELA) process used with amorphous silicon in the low-temperature polycrystalline silicon (LTPS) transistor manufacturing process [11].

To separate the polyimide and the carrier substrate, the wavelength of the excimer laser irradiation must be in the ultraviolet (UV) range, which is the light absorption region of the polyimide. When the excimer laser is irradiated on the PI, the PI structure is broken, and gas is generated by thermal decomposition. The carrier substrate and the PI film are separated by this gas. In order to completely separate the carrier substrate and the PI film, the excimer laser fluence is an important parameter [12]. If the laser fluence rate is too high, the PI film will tear or deform, and if it is too low, it will not separate from the carrier substrate [13].

Excimer lasers have the advantage of being able to easily produce high-power beams, but they require very expensive gases such as chlorine (Cl_2_) and xenon (Xe), and they use expensive optical amplifiers and highly reflective mirrors, resulting in a high facility investment cost. Since expensive consumable parts are required, considerable cost is also required for maintenance. Crucially, because LLO technology uses a line scan method, it is difficult to speed up for large-scale substrates and mass production [14].

Core technologies employed in LLO technology are surface-free technology by laser annealing (SUFTLA) and electronics on plastic (EPLaR) technologies [15,16,17]. SUFTLA technology is a lift-off technology that used a-Si:H as a sacrificial layer and is performed as follows. When the sacrificial layer is deposited using plasma enhanced chemical vapor deposition (PECVD), hydrogen is generated by SiH_4_ gas used for deposition. Then, when the laser beam is irradiated on the sacrificial layer, the bond between hydrogen and silicon is broken, and hydrogen gas is generated, which separates the glass substrate. Another technology, EPLaR, is the most basic lift-off technology for coating PI on a carrier substrate and irradiating with a laser [17]. However, since both methods use an expensive excimer laser source and an optical system for complex ultraviolet lasers, initial equipment and maintenance costs are very high. Therefore, it is necessary to develop a novel approach for the potential possibility of reduction of manufacturing cost and improvement of productivity.

In this study, we propose development of new lift-off process technology by optimizing the main process parameters of xenon flash lamp lift-off (XF-LO) technology to realize a non-laser method, XF-LO technology. We investigate and describe the basic principles of XF-LO technology.

## 2. Experimental Details

### 2.1. Materials

The light to heat conversion layer (LTHC), which converts light energy into thermal energy, is a core component of XF-LO technology. XF-LO technology is based on the principle of exfoliation, where heat is generated in the LTHC coating between a carrier substrate and a polyimide film by irradiating light. The working principle of LTHC is as follows. When light is irradiated on the LTHC, maximum efficiency of light absorption occurs under optimal conditions of optical interference and absorption and dispersion of the metal layer.

The LTHC is a resonant-based, lithography-free, broadband, polarization-independent optical absorber based on a three-layer ultrathin film [18]. The LTHC is made of a total of four layers: the first and second metal layers, a buffer layer and a barrier layer. The roles of each layer are as follows. The first metal layer is a thin metal layer. It is the first layer reached by xenon flash lamp (XFL) irradiation during the lift-off process. This layer transmits, reflects, and absorbs light. Next is a second thick metal layer that mainly serves as a reflector, followed by a buffer layer, which is a phase-matching layer to adjust the phase of light reflected from the second metal layer. Finally, a barrier layer serves to transfer heat without allowing direct contact between the PI film and heat generated in the second metal layer. Although the function of the LTHC can be performed without a barrier layer, the damage can be caused by direct contact between the PI film and metal layer, so an additional barrier layer was used.

**Figure 1:**
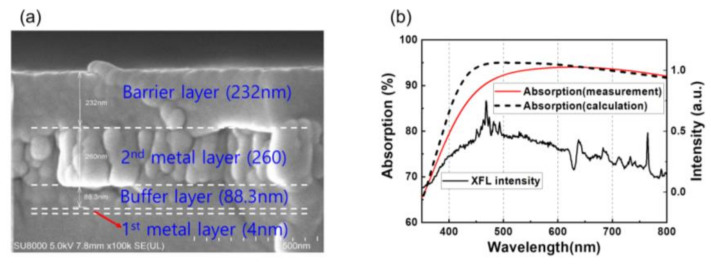
(**a**) SEM image of light to heat conversion layer (LTHC); (**b**) calculation and measurement data of xenon flash lamp (XFL) absorbed by the LTHC.

Optical calculations were performed to determine the optical properties of thin multilayer films, using commercial software SETFOS 4.3 (Fluxim AG, Winterthur, Switzerland). The refractive index (n) and extinction coefficient (k) of molybdenum (Mo) and silicon dioxide (SiO_2_) were obtained using SETFOS 4.3. The thickness of the first metal layer and buffer layer (SiO_2_) and the second metal layer were optimized to 4, 90, and 300 nm. Based on simulation results, Mo was used as the material for the first and second metal layers, and SiO_2_ was used as the material for the buffer layer and barrier layer. To fabricate the LTHC layer, the Mo layer for the first and second metal layers was deposited by an electron-beam (E-beam) evaporator, and the SiO_2_ layer for the buffer and barrier layer was deposited by plasma enhanced chemical vapor deposition (PECVD). However, when the thickness of the buffer layer (SiO_2_) and second metal layer (Mo) were observed by scanning electron microscopy (SEM), they were determined to be 88.3 and 260 nm, respectively, as shown Figure 1a. Figure 1b is a graph showing calculation and measurement of light absorption in the LTHC layer and spectrum of XFL intensity. XFL intensity shows a broad band spectrum, ranging from approximately 400 to 800 nm. This spectrum of visible light matches the spectrum of light absorption by the LTHC layer well. High levels of light absorption, 94.9 % (calculated at 550 nm) and 93.5 %, were observed.

The polymerization reaction with polyamic acid (PAA) was conducted based on the total weight of 100 g, and polymerization was carried out by holding the reaction vessel in a nitrogen atmosphere and maintaining the initial reaction temperature at 0 °C for 2 h. The basic lift-off characteristics were checked by changing the diamine monomer based on pyromellitic dianhydride (PMDA), and it was confirmed that there was a difference in surface damage to the film after the lift-off process, depending on the fluorine content of the PAA. Therefore, a polyimide prepared using PAA with a high fluorine content of PMDA/2,2’-bis(trifluoromethyl)benzidine was used in this study. As mentioned above, XF-LO technology does not use an excimer laser in LLO technology but instead uses a xenon flash lamp (XFL), so the XF 12100LCW (Unilam Co., Ltd., Ulsan, Republic of Korea) was used as the light source. The light energy (2.61, 4.20, 5.80, and 7.40 J/cm^2^) was varied by adjusting the exposure time (3, 5, 7, and 9 ms) with a fixed operating voltage of 500 V. Additionally, the light intensity of XFL is shown in Figure 1b.

### 2.2. Fabrication

**Figure 2:**
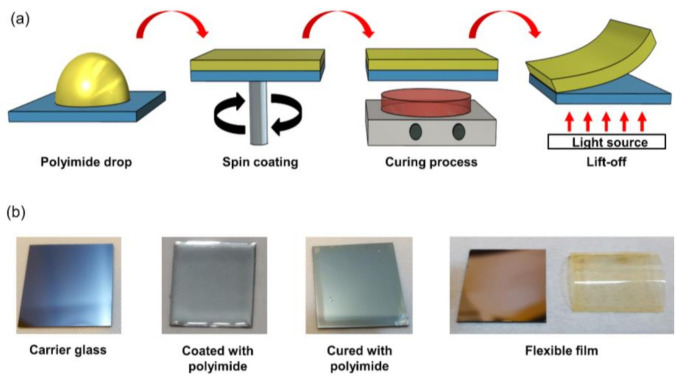
(**a**) A schematic description of fabricate flexible film; (**b**) an image of fabricated polyimide (PI) film.

Figure 2a shows a schematic description of the process for manufacturing the PI film. The process sequence is as follows. First, the PI solution is dropped on the carrier substrate (25 × 25 mm) coated with LTHC. Second, a spin-coating process is performed in which the PI solution is evenly coated on the carrier substrate. Third, after completing the spin-coating process, a curing process is performed for imidization of the PI solution. Since PI characteristics can be affected by curing process conditions, the process was performed under optimized conditions [19]. In this study, the PI-coated substrate was pre-baked at a temperature of 100 °C for 30 min, cured at 200 °C for 8 min and then cured at 350 °C for 20 min. Finally, a light source in the form of a xenon flash lamp was irradiated onto the substrate to detach the PI film from the carrier substrate to complete the flexible film. Figure 2b shows an image of the fabricated PI film.

**Figure 3:**
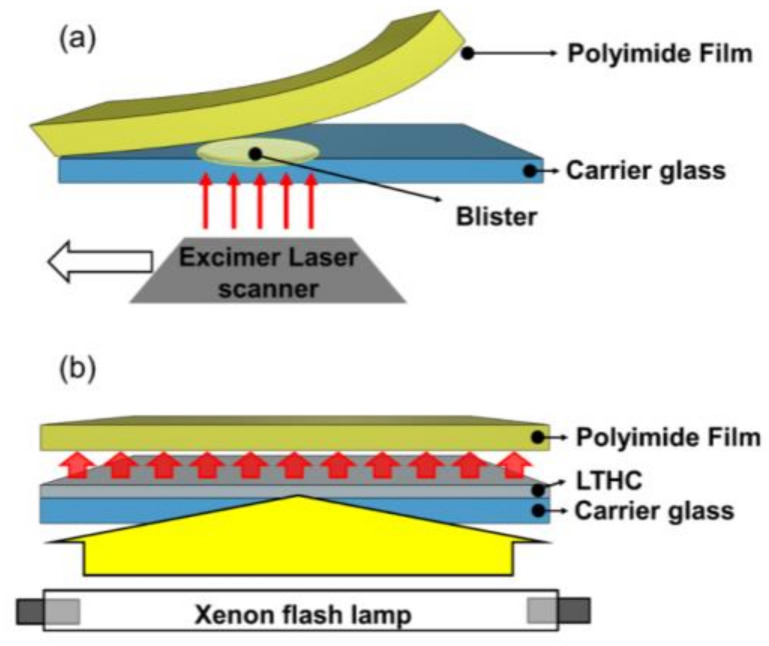
Schematic description of the lift-off process: (**a**) laser lift-off process and (**b**) xenon flash lamp lift-off (XF-LO) process.

Figure 3 is a schematic description of lift-off technologies LLO and XF-LO. In the LLO process shown in Figure 3a, the excimer laser is irradiated on the bonding surface of the PI and carrier substrate, which is separated by the generated gas. The amount of gas generated depends on laser fluence, which determines the size of the blister. If the blister size is too small, the PI and carrier substrate cannot be lifted-off. If the blister size is too large, cracks will occur in the PI film. Additionally, since it is a line scan method, processing time is limited, and increasing the size of the substrate is currently difficult to realize. In contrast, XF-LO technology shown in Figure 3b uses the heat generated by irradiating the XFL on the LTHC to be lifted-off. Because it uses the area scan method, it is a technology that can reduce time and increase the size of substrate.

### 2.3. Methods

#### 2.3.1. SEM Analysis

In order to measure the thickness of the PI film that could be completely lifted-off without damage using XF-LO technology, five samples with different spin-coating conditions were prepared.

#### 2.3.2. Thermal Gravimetric Analysis (TA Instruments, TGA Q500)

Thermal gravimetric analysis (TGA) was performed to analyze stripping characteristics of the PI films according to the energy irradiated by the XFL during XF-LO technology. To evaluate the thermal stability of the PI film, 5 wt% weight reduction temperatures were measured via TGA.

#### 2.3.3. FTIR Analysis (Thermo Fisher Scientific, Nicolet iS50 FTIR Spectrometer)

FTIR measurements were taken for film surface analysis after PI film stripping. FTIR transmittance graphs were obtained and analyzed up to 600 to 2000 cm^−1^ wave numbers.

#### 2.3.4. Transmittance Analysis (Sninco, UV–VIS Spectrophotometer, Mega-800).

In order to use the produced PI film as a flexible electronic device, transmittance of the film is also an important characteristic. Permeability of the PI film was measured to analyze the effect of XFL energy strength on the surface of the PI film.

## 3. Results and Discussion

**Figure 4:**
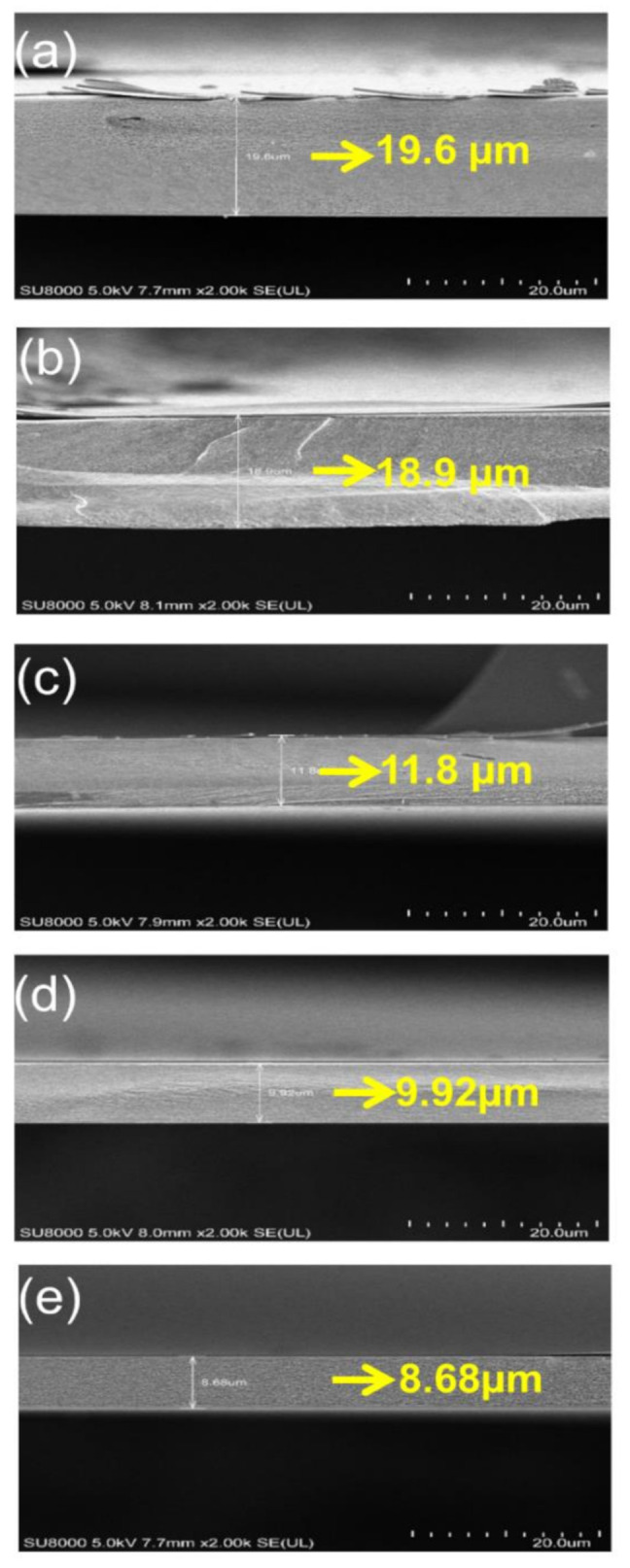
SEM image of the PI film after coating the inorganic barrier layer, SiO_2_ and SiNx, according to the PI spin-coating conditions: (**a**) 2000 rpm, 60 s; (**b**) 2000 rpm, 120 s; (**c**) 3000 rpm, 120 s; (**d**) 4000 rpm, 120 s and (**e**) 5000 rpm 120 s.

Figure 4a is an SEM observation of a substrate PI spin-coated for 60 s at 2000 rpm. The PI film was coated to a thickness of 19.6 µm. Figure 4b shows a PI that was spin-coated at 2000 rpm for 120 s, coated to a thickness of 18.9 µm. Figure 4c was spin-coated at 3000 rpm for 120 s, Figure 4d was 4000 rpm for 120 s and Figure 4e was 5000 rpm for 120 s. Films with different spin-coating speeds produced controlled thicknesses of 11.8, 9.92 and 8.68 µm. Here, to prepare samples without damage for SEM observation, 200 nm thickness SiNx was deposited on the PI film.

**Figure 5:**
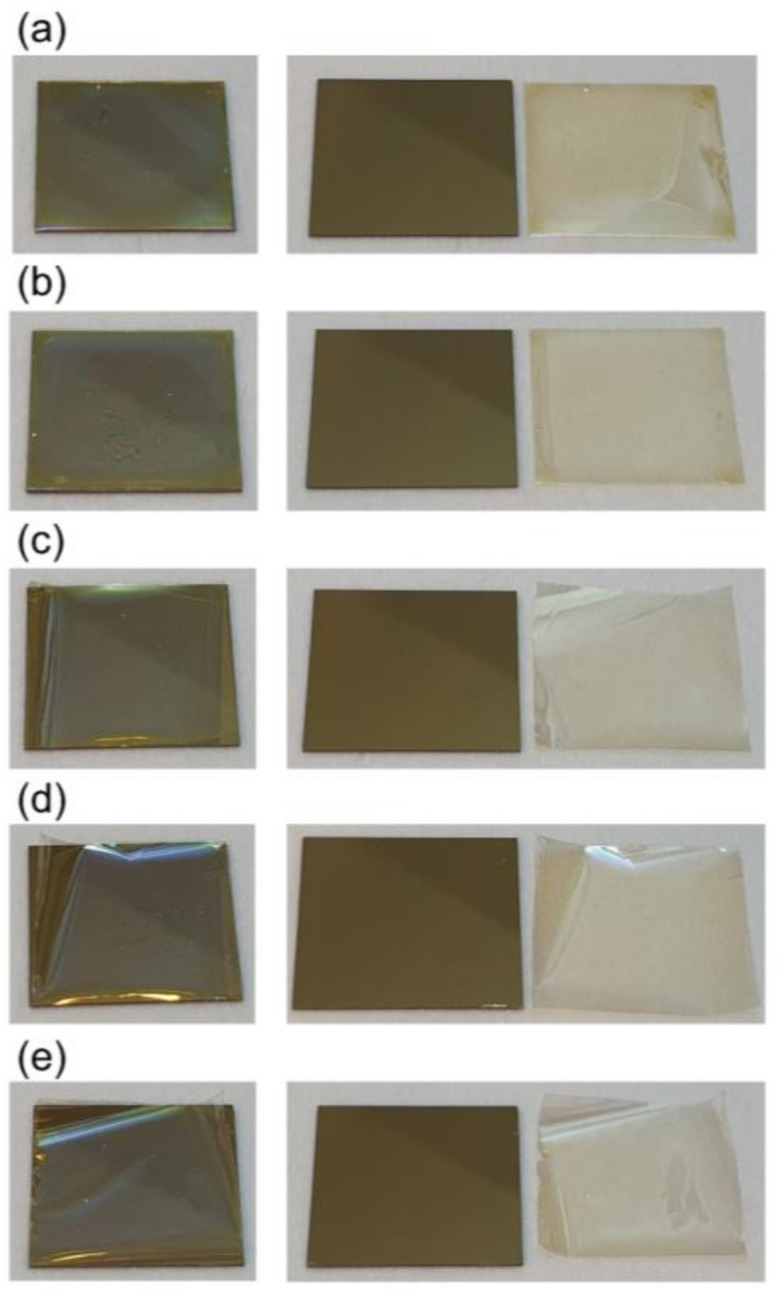
Image of the lift-off PI film according to the PI spin-coating conditions: (**a**) 2000 rpm, 60 s; (**b**) 2000 rpm, 120 s; (**c**) 3000 rpm, 120 s; (**d**) 4000 rpm, 120 s and (**e**) 5000 rpm, 120 s.

Figure 5 shows lift-off results of the PI film according to the spin-coating conditions. Carrier substrates having different thicknesses of PI film were irradiated by XFL light and then the PI films were peeled off. After lift-off, it was determined PI films from 19.6 to 8.68 µm thick could be lifted off without cracking. The PI film had a slight crease, but lift-off was possible without cracking to a thickness of 8.68 µm.

In the lift-off process of the thin film, it is not only important to release the PI film completely from the carrier substrate, but it is also important to prevent damage to the PI film [11]. With conventional LLO technology, lift-off characteristics vary according to the intensity of the laser that releases the film during the lift-off process. If the laser strength is too weak, the PI film will not be completely released from the carrier substrate and too strong will cause damage to the PI film. For XF-LO technology, lift-off characteristics were confirmed by varying the energy intensity of the XFL used to separate the PI film from the carrier substrate. The PI film used in the experiment was manufactured under the conditions listed in Figure 5b with a thickness of 18.9 µm for process stability. In addition, XFL energies were varied, with increasing pulse width from 3 to 9 ms with a fixed pulse height of 500 V. The pulse widths of 3, 5, 7, and 9 ms correspond to energies of 2.61, 4.2, 4.2, and 4.2 J/cm^2^.

**Figure 6:**
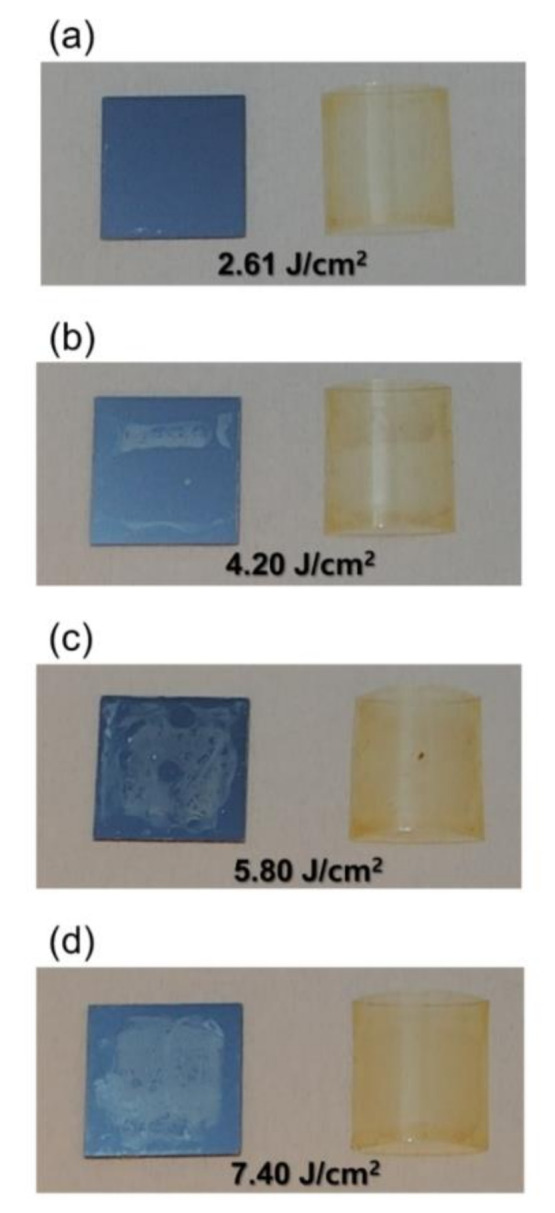
Lift-off characteristics according to XFL energy: (**a**) 2.61; (**b**) 4.20; (**c**) 5.80 and (**d**) 7.40 J/cm^2^.

Some research works show that a higher photonic energy causes dissociation of PI films or increase in adhesion to the substrate [20,21]. In this work, light energy penetrating the LTHC is almost nothing due to the high thickness of the second Mo layer in the range of about 260–300 nm. As shown in Figure S1 of the Supplementary Materials, the transmittances at 550 nm wavelength were 0.000000033% (calculation) and 0.000433601% (measurement), respectively. Therefore, only thermal energy has influence on the lift-off process of the PI films. When intense pulsed light (IPL) from XFL arrives at the LTHC, the absorbed light is converted into heat in a flash, and then the PI film is separated due to this instant thermal energy. In this work, it was confirmed that the PI film was completely lifted-off from the carrier substrate at the energy of 2.61 J/cm^2^. Figure 6 shows the PI film and carrier substrate that were used to perform the lift-off process with different XFL energy intensities. Figure 6a shows that the XFL energy was 2.61 J/cm^2^ at lift off, and it can be seen that the PI film and the carrier substrate are completely released. However, in Figure 6b with XFL energy of 4.2 J/cm^2^, soot occurred on the carrier substrate. In addition, in Figure 6c,d, with stronger energy intensities, the soot phenomenon was more clearly observed. When the XFL energy was 2.61 J/cm^2^ or more, the film and the carrier substrate were completely lifted-off, but when the lift-off process was performed with more energy, the heat generated from the LTHC overloaded the PI film, causing a combustion phenomenon. It seemed that soot was produced on the carrier substrate and the PI film. Due to this kind of combustion phenomenon, we think the PI film could be peeled off. However, the exact mechanism by which the PI film peels during this process is currently unknown.

**Figure 7:**
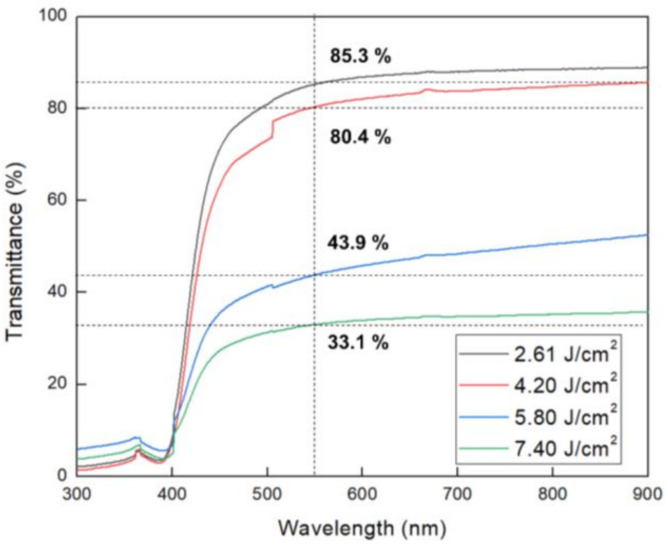
Variation of the obtained transmittance with XFL energy.

The transmittance of the PI film was measured in order to check whether the soot to the carrier substrate during the XF-LO process affected transmittance of the PI film. Figure 7 is a graph measuring transmittance according to the pulse width of XFL. Transmittance of 550 nm was measured for each sample. As a result of transmittance measurements, the XFL energy is 85.3% at 2.62 J/cm^2^, 80.4% at 4.20 J/cm^2^, 43.9% at 5.80 J/cm^2^ and 33.1% at 7.40 J/cm^2^, as the XFL energy intensity increases were confirmed to decrease. The reason for this is that when the irradiated XFL energy increases during the XF-LO process, more heat is generated in the LTHC than necessary, deforming the PI film structure and causing it to stick to the carrier substrate. As the PI film structure is deformed and the surface is roughened, transmittance is reduced and also lowered when the sticking phenomenon occurs. To further understanding the thermal durability of the PI films, TGA analysis was conducted as the XFL energy has increased.

**Figure 8:**
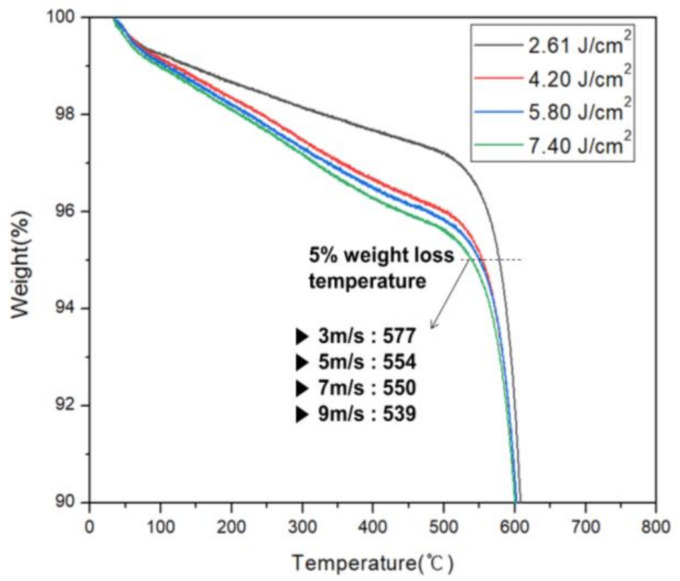
Variation of the obtained TGA with the XFL energy.

TGA results confirmed the effect of XFL energy intensity on thermal durability of the PI film. In TGA evaluation, a thermogravimetric curve was obtained at a heating rate of 10 °C/min, and the T_d_5 wt% was measured. In this case, T_d_5 wt% is the temperature at which 5% weight reduction occurs when heat is applied to the PI film. Figure 8 is the TGA graph. When XFL energy was 2.61 J/cm^2^, T_d_5 wt% was 577 °C, which was the most stable thermal durability. Thermal instability was confirmed as energy intensity increased. When XFL energy was 4.20 J/cm^2^, T_d_5 wt% was 554 °C when the 5.80 J/cm^2^ was 550 °C and when the 7.40 J/cm^2^ was 539 °C. These effects are believed to originate in the LTHC, which radiates more heat than necessary. When XFL energy increases, the temperature of the heat radiated from the LTHC increases, burning the PI film and reducing thermal durability of the PI film, resulting in structural changes.

Accordingly, the lift-off method was judged to be effective when the energy intensity of the XFL was less than 2.61 J/cm^2^ during the XF-LO process. However, TGA is a quantitative analysis method that only reveals the rate at which the weight decreases with temperature, and it is not possible to know which substances are decomposed and which substances are generated. To investigate this, qualitative analysis was performed on the phenomenon occurring in the PI film during the XF-LO process, using FTIR measurement.

**Figure 9:**
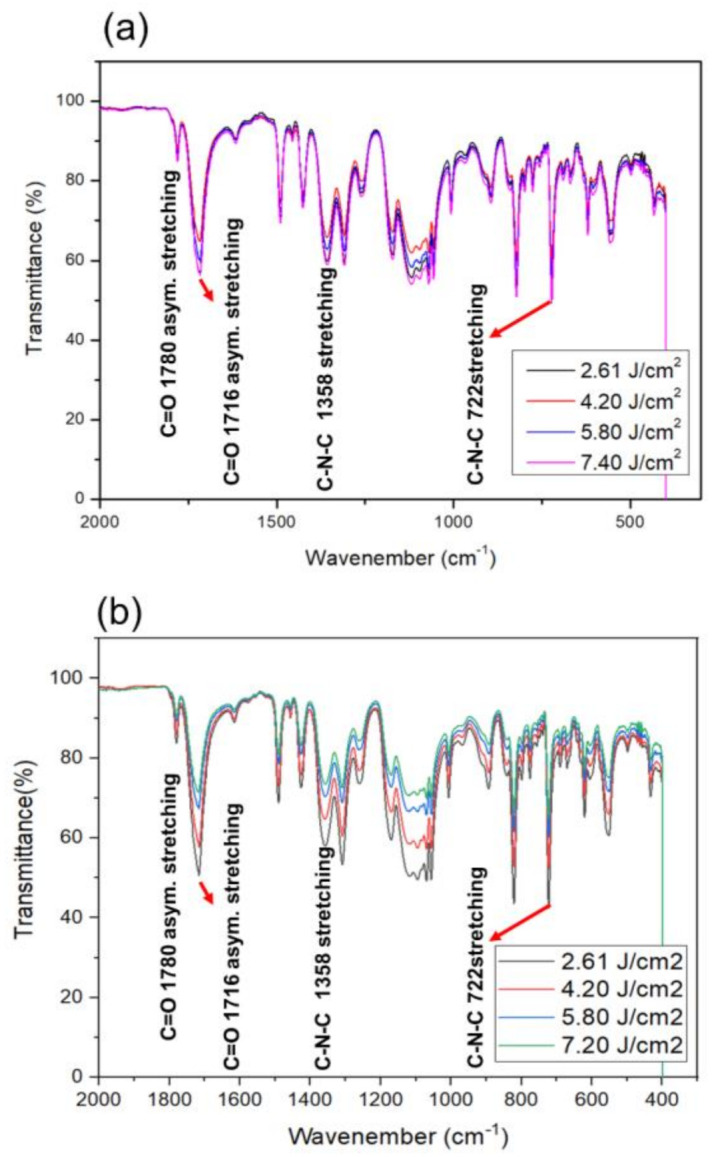
Measurement of FTIR to observe the change of film surface according to XFL energy: (**a**) top side and (**b**) bottom side.

Figure 9 shows FTIR measurement data of the PI film lift-off for XF-LO technology. Peaks corresponding to C=O asymmetric stretching (1780 cm^−1^), C=O symmetric stretching (1716 cm^−1^), C-N stretching (1358 cm^−1^) and cyclic C=O bending (722 cm^−1^) of the imide structures were observed [22,23,24]. To check the change in release surface caused by heating during the lift-off process, not only the release surface of the PI film but also the FTIR of the upper side were measured. Figure 9a is the FTIR measurement result of the upper side of the PI film, not the release side. Since damage does not occur on the surface where the LTHC is not in direct contact, no change in transmittance intensity was observed. However, Figure 9b on the bottom side, which is the release surface between the carrier substrate and the PI, shows transmittance intensity decreased as XFL energy intensity increased.

As with the TGA result, it was determined that as the pulse width is increased, combustion occurs on the release surface of the PI film and damage occurs, resulting in a change in the PI structure. In addition, PI film that has undergone combustion exhibits uneven adhesion as it is released from the carrier substrate and forms nanopillar shapes which increase surface roughness. When this occurs, deeply pitted spaces occur and IR transmittance intensity decreases. When XFL energy intensity increases, thermal durability of PI film also decreases. In addition, it was observed that combustion occurs when thermal durability decreases and the PI structure is deformed, resulting in an increase in surface roughness and a decrease in transmittance. The combined results confirmed that when the XF-LO process was performed at the optimized threshold XFL energy of 2.62 J/cm^2^, it was possible to process an ultra-thin PI film of up to 10 µm or less, but more XFL energy deteriorated the process characteristics. These findings demonstrate that the optimized XF-LO process is practical for manufacturing flexible electronic devices.

## 4. Conclusions

Lift-off technology is the core process for fabricating next-generation displays in a scale sufficient to realize large-area flexible electronic devices. Although LLO technology is currently dominant, its use is limited by high process costs. This study was conducted to investigate development of XF-LO, a non-laser lift-off technology. LTHC, which is used as a core component, converts light into thermal energy when the incident light of XFL arrives at the LTHC. Tests confirmed that the PI film could be separated from the carrier substrate using this method. In addition, it was confirmed that a PI film of at least 8.68 µm can be completely separated without scarring. However, as the XFL energy became stronger, the PI film burned and the structure was deformed. To solve this problem, the process was investigated for various energy intensities and was found to be optimized at a threshold energy of 2.61 J/cm^2^.

## Supplementary Materials

The following are available online at https://www.mdpi.com/2072-666X/11/11/953/s1, Figure S1. Measured and calculated spectra of transmittance, reflectance and absorption of LTHC layer.
