# Supplementary material for: Xenon Flash Lamp Lift-Off Technology without Laser for Flexible Electronics
Source: Micromachines (Basel). 2020 Oct 22;11(11):953. doi: 10.3390/mi11110953 (PMC7690583; doi:10.3390/mi11110953)
Supplement: Supplementary file 1 [file micromachines-11-00953-s001.pdf]

micromachines

*Article*

# Xenon Flash Lamp Lift-off Technology without Laser for Flexible Electronics

**Sang Il Lee, Seong Hyun Jang, Young Joon Han, Jun yeub Lee, Jun Choi* and Kwan Hyun Cho***

[1] Manufacturing Process Platform R&D Department, Korea Institute of Industrial Technology (KITECH), 15588, Ansan, Korea; twosangone@kitech.re.kr (S.I.L.); youngjhan@kitech.re.kr (Y.J.H.); june522@kitech.re.kr (J.Y.L.)

[2] Human Convergence Technology R&D Department, Korea Institute of Industrial Technology (KITECH), 15588, Ansan, Korea; seonghyun@kitech.re.kr (S.H.J.)

* Correspondence: skywork1@kitech.re.kr (J.C.); khcho@kitech.re.kr (K.H.C.)

Received: 16 September 2020; Accepted: 20 October 2020; Published: 22 October 2020

## 1. Supplementary Materials

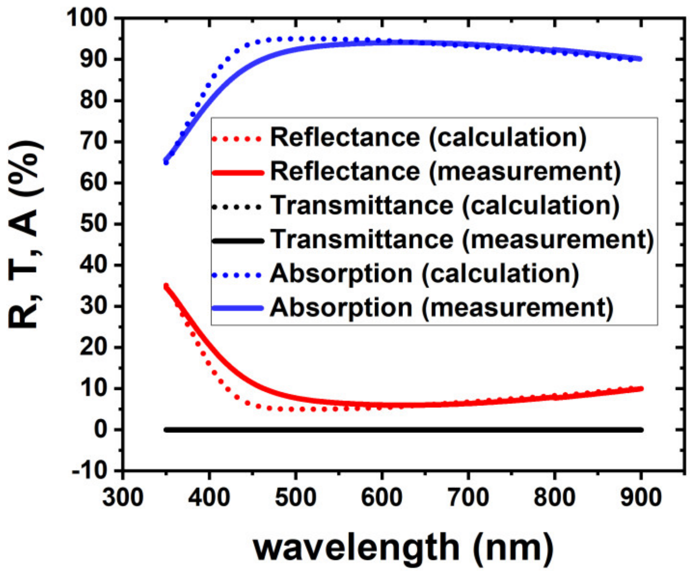


**Figure S1.** Measured and calculated spectra of transmittance, reflectance, and absorption of LTHC layer.

© 2020 by the authors. Licensee MDPI, Basel, Switzerland. This article is an open access article distributed under the terms and conditions of the Creative Commons Attribution (CC BY) license (http://creativecommons.org/licenses/by/4.0/).
